# An intranasal vaccine targeting the receptor binding domain of SARS-CoV-2 elicits a protective immune response

**DOI:** 10.3389/fimmu.2022.1005321

**Published:** 2022-11-16

**Authors:** Li Chen, Haiwei Zhang, Moxuan Li, Bihao Wu, Zhe Zhang, Rui Gong

**Affiliations:** ^1^ CAS Key Laboratory of Special Pathogens and Biosafety, Wuhan Institute of Virology, Center for Biosafety Mega-Science, Chinese Academy of Sciences, Wuhan, Hubei, China; ^2^ University of Chinese Academy of Sciences, Beijing, China; ^3^ Hubei Jiangxia Laboratory, Wuhan, Hubei, China

**Keywords:** SARS-CoV-2, intranasal vaccine, RBD-Fc, mucosal immunity, mucosal vaccine

## Abstract

Severe acute respiratory syndrome coronavirus 2 (SARS-CoV-2), the pathogen responsible for COVID-19, has caused an ongoing worldwide pandemic. Due to the rapid emergence of variants of concern (VOCs), novel vaccines and vaccination strategies are urgently needed. We developed an intranasal vaccine consisting of the SARS-CoV-2 receptor binding domain (RBD) fused to the antibody Fc fragment (RBD-Fc). RBD-Fc could induce strong humoral immune responses *via* intranasal vaccination. Notably, this immunogen could efficiently induce IgG and IgA and establish mucosal immunity in the respiratory tract. The induced antibodies could efficiently neutralize wild-type SARS-CoV-2 and currently identified SARS-CoV-2 VOCs, including the Omicron variant. In a mouse model, intranasal immunization could provide complete protection against a lethal SARS-CoV-2 challenge. Unfortunately, the limitation of our study is the small number of animals used in the immune response analysis. Our results suggest that recombinant RBD-Fc delivered *via* intranasal vaccination has considerable potential as a mucosal vaccine that may reduce the risk of SARS-CoV-2 infection.

## Introduction

The COVID-19 pandemic, caused by severe acute respiratory syndrome coronavirus 2 (SARS-CoV-2) (1, 2), is ongoing worldwide and has resulted in devastating consequences to human health and the global economy. To date, the WHO has reported hundreds of millions of COVID-19 cases globally and more than 6 million deaths (https://covid19.who.int/).

SARS-CoV-2, a member of the coronavirus family, is a large, enveloped, single-stranded, and positive-sense RNA virus. The 30 kb genome of SARS-CoV-2 encodes four major structural proteins, including the nucleocapsid protein, envelope protein, membrane protein and spike protein (S protein), as well as 16 nonstructural proteins (1). The S protein is a highly glycosylated class I viral fusion protein that forms a trimer on the SARS-CoV-2 virion. It contains two basic functional subunits, receptor-binding subunit S1 and membrane-fusion subunit S2 (3–6). SARS-CoV-2 enters host cells by binding to the human angiotensin-converting enzyme II (hACE2) cellular receptor through an internal receptor binding domain (RBD) in the S1 subunit (7–9), which is followed by fusion of the viral and cellular membranes *via* a heptad repeat in the S2 subunit (5). Through epitope mapping, the RBD was identified as a major target of neutralizing antibodies (3, 10–13) and dominant T-cell epitopes (14), suggesting that the RBD is a promising primary candidate for SARS-CoV-2 vaccine development.

The urgent need for an effective vaccine to control and end the global pandemic has revolutionized vaccine technology and stimulated the investigation of multiple vaccine candidates. In fact, more than 800 vaccines are undergoing clinical evaluation, of which approximately 47 have been approved for clinical use by the WHO (https://covid19.trackvaccines.org/), including mRNA vaccines, inactivated vaccines, viral vector vaccines and protein subunit vaccines (15). However, breakthrough infections of fully vaccinated individuals have been reported worldwide (16). It seems that these approved vaccines might primarily decrease mortality rather than prevent infection (17–20). In addition, immune escape and the persistent emergence of new variants of concern (VOCs) (e.g., Omicron and its sublineages) have seriously challenged the effectiveness of the currently approved vaccines (21–25).

Since SARS-CoV-2 mainly enters the human body from the proximal to distal respiratory tract (26), an increase in the protective immune response in the respiratory tract is highly desirable. However, the majority of current approved vaccination strategies involve intramuscular injection, which mainly induces an immune response in the blood but not in the respiratory tract (27–30). The antibody Fc fragment is a dimeric protein that has important effects on the properties of Fc-fusion proteins, including improvements in physicochemical properties, modulation of immunogenicity, and extension of serum half-life (31). Notably, Fc-fusion proteins used as vaccines can activate the mucosal immune system by binding to the neonatal Fc receptor (FcRn) (31, 32). In our previous study, two potent neutralizing antibodies were identified from a phage library using RBD-Fc as an antigen (33). Here, we further evaluated RBD-Fc as a candidate subunit vaccine against SARS-CoV-2 *via* nasal vaccination.

In this research, we show that RBD-Fc formulated with alum as an adjuvant could induce robust humoral immune responses. Importantly, intranasal immunization results in the preferential induction of IgA and the establishment of mucosal immunity in the respiratory tract. The sera from immunized mice could efficiently neutralize wild-type SARS-CoV-2 (SARS-CoV-2 WT) and variants, including the emerged Omicron variant. Moreover, fully vaccinated mice were completely protected against challenge with SARS-CoV-2 WT. Collectively, these results suggest that recombinant RBD-Fc has considerable potential as a mucosal vaccine that could provide protective immune response against viral infection and may further reduce the risk of SARS-CoV-2 infection.

## Methods

### Cells and viruses

Vero E6 (catalog no. GDC146, CCTCC) and 293T cells (catalog no. GDC187, CCTCC) were maintained in DMEM (Gibco) supplemented with 10% FBS (Gibco) 100 U/mL penicillin and 0.1 mg/mL streptomycin (Gibco). HEK293F cells (Thermo Fisher Scientific) were grown in suspension and cultured in Freestyle 293 expression medium (Invitrogen). Authentic SARS-CoV-2 (strain: IVCAS 6.7512) was obtained from the National Virus Resource, Wuhan Institute of Virology, Chinese Academy of Science. SARS-CoV-2 was passaged on Vero E6 cells at a multiplicity of infection of 0.05. The virus-containing cell culture medium was harvested after 48 hours of infection and stored at -80°C. All processes in this study involving authentic SARS-CoV-2 were performed in a biosafety level 3 (BSL-3) facility.

### Mouse experiments

Female 4- to 6-week-old BALB/c mice were purchased from Beijing Vital River Laboratory Animal Technology Co. Ltd. Female 4-week-old K18-hACE2 transgenic mice were purchased from GemPharmatech Co. Ltd. All animals were randomly divided into groups and housed in specific pathogen-free animal care facilities at the Animal Center of the Wuhan Institute of Virology, Chinese Academy of Science. Standard procedures were used to maintain animals. Viral infections were performed in a BSL-3 facility. All processes in the animal experiments were in line with recommendations for the care and use of laboratory animals and the Institutional Review Board of the Wuhan Institute of Virology, Chinese Academy of Science (Ethics number: WIVA34202103).

### Purification of RBD and hACE2 recombinant protein

The optimized sequence of the SARS-CoV-2 RBD (GenBank: QHR63250.2, residues R319–F541) was cloned into the pCAGGS expression vector, with a mouse IgG2a Fc fragment at the C-terminus. The plasmid containing the chimeric RBD-mFc fragment was verified by DNA sequencing. The coding sequence of hACE2-Fc (GenBank: Q9BYF1, residues M1–S709) was inserted into the expression vector pCAGGS with a human IgG Fc fragment at the C-terminus. HEK293F cells were transiently transfected with these plasmids using polyethyleneimine (PEI-25 kDa, Polysciences). After 5 days of culture, the supernatants were collected, and the soluble proteins were purified by protein A resin (GE Healthcare). The RBD construct (R319–F541) in pCAGGS with a His tag at the C-terminus was expressed and purified from HEK293F cells through Ni-NTA agarose. These purified proteins were concentrated and exchanged into PBS using a 10-kDa centrifugal filter device (Millipore). The concentrations of the purified proteins were determined by a NanoPhotometer N60 (Implen) according to the corresponding extinction coefficient. Coomassie blue staining and size exclusion chromatography were performed to confirm the purity of the recombinant proteins.

### Binding of recombinant RBD proteins to hACE2-Fc

For validation of biological activity, the recombinant RBD-mFc and RBD proteins were coated on high-bind 96-well plates (Corning) at 4 μg/mL overnight at 4°C and blocked with PBS containing 3% skim milk (Bio-Rad) at 37°C for 1 hour. The plates were washed with PBST (PBS containing 0.05% Tween-20) three times and serially diluted hACE2-Fc was added and incubated at 37°C for another 1.5 hours. After five washes with PBST, HRP-conjugated goat anti-human IgG Fc antibody (1:5000, Abcam) was used as a secondary antibody. After 1 hour of incubation, the plates were washed with PBST five times. The binding was measured with the subsequent addition of the substrate diammonium 2,2-azinobis (3-ethylbenzothiazoline-6-sulfonate) (ABTS; Life), and the absorbance signal was read at 405 nm using a microplate reader (BioTek). The half-maximal effective concentration (EC_50_) was calculated using GraphPad Prism (version 8, GraphPad).

### Plasma half-life of recombinant protein through intranasal administration

Following anesthesia with 2% isoflurane, six BALB/c mice were divided into two groups: RBD-mFc and RBD. Mice in the two groups were intranasally injected with RBD-mFc and RBD proteins in a 20 μL volume (50 μg each). Blood samples were collected at 0.5, 1, 2, 6, 12, 24, 36, 48, 60, 72, 84, and 96 hours from each mouse after administration. nCoVmab1 (33), a monoclonal antibody to RBD, was coated onto 96-well plates. Known concentrations (determined by measurement at A280) of RBD-mFc and RBD proteins were used to generate a standard curve. The absorption values of each sample were transferred into concentrations based on a standard curve.

### Optimization of the dose of immunization

To optimize the dose for immunization, sixteen BALB/c mice were used and randomly divided into four groups. Each mouse was intranasally immunized with different doses of RBD-mFc. Specifically, 1, 5, 10 and 20 μg doses of immunogens were formulated with equal volumes of Imject™ Alum Adjuvant (Thermo Fisher Scientific) in a 20 μL total volume. Serum was collected at weeks 0, 2, 4 and 6. Mice in the 10 μg RBD-mFC group were observed over time to analyze the durability of the IgG and IgA antibodies in serum, with blood samples collected every month after the booster immunization.

### Immunization of BALB/c mice

For BALB/c vaccination, all proteins were mixed with an equal volume of Imject™ Alum Adjuvant (Thermo Fisher Scientific) according to the manufacture's instructions. Briefly, the capped bottle of Imject Alum was shaken well, and then Imject Alum was added dropwise with constant mixing to the immunogen solution at a volume ratio of 1:1. Each mouse in the RBD/i.m. group (n=4) was immunized by intramuscular injection in the thigh of 20 μg RBD-mFc protein delivered in a total volume of 50 μL. Mice in the RBD/i.n. group (n=4) were inoculated intranasally with an equal dose of RBD in a 20 μL volume (20 μg per mouse), and mice in the RBD-mFc/i.n. group (n=4) were intranasally immunized with RBD-mFc in a 20 μL volume (20 μg per mouse), with equal amounts in each group. Equal volumes of adjuvant mixed with PBS-vaccinated mice were intranasally immunized and used for comparison. All mice were immunized at two-week intervals in a homogeneous prime-boost-boost scheme, and blood samples were collected from the ophthalmic vein every two weeks. Serum was separated by centrifugation and stored at -80°C until analysis. Upon completion of vaccination, all mice were humanely euthanized with an overdose of sodium pentobarbital (75 mg/kg). Blood samples were collected and serum was centrifuged. Bronchoalveolar lavage (BAL) fluid from all mice was collected by flushing the lungs using 0.5 mL of sterile PBS *via* a tracheal cannula. The trachea and nose from all mice were removed and washed with 0.2 and 0.5 mL of sterile PBS, respectively. The BAL fluid and washes were centrifuged, and the supernatants were collected and stored at -80°C until needed.

### Detection of RBD-specific antibodies

For the detection of RBD-specific antibodies in the serum, BAL, nasal wash and tracheal wash, RBD protein was coated onto 96-well high binding microplates (Corning) at 4 μg/mL overnight at 4°C. After washing three times with PBST, the plates were blocked with PBS containing 3% skim milk (Bio-Rad) at 37°C for 1 hour. Again, the plates were washed with PBST three times and pat dried. Twofold serially diluted samples were added and incubated for 90 min. After washing five times, HRP-conjugated anti-mouse IgG (1:10000, Abcam), IgA (1:2000, Abcam) or IgG subclass-specific antibodies (1:10000, Abcam) were used as secondary antibodies. After the addition of ABTS, the absorbance signal was read at 405 nm using a microplate reader. The endpoint titer was determined as the reciprocal of the highest dilution showing an absorbance value that was 2-fold greater than the background level.

### Intracellular cytokine staining and flow cytometry

Intracellular cytokine staining (ICS) was performed on splenocytes harvested from vaccination-treated BALB/c mice. In detail, two weeks after the second boost immunization, mice in PBS (n=4), RBD/i.n. (n=4), RBD-mFc/i.n. (n=4) and RBD-mFc/i.m. (n=4) were euthanized and spleens were harvested. The splenocytes were seeded into the plates at 1×10^6^ cells/well and stimulated with SARS-CoV-2 RBD protein at 20 μg/mL for 42 hours (37°C, 5% CO_2_). Phorbol 12-myristate 13-acetate (PMA, 25 ng/mL) plus ionomycin (500 ng/mL) was used as a positive control, and complete medium alone was used as a negative control. During the last 6 hours, brefeldin A (BFA) was added at 10 μg/mL. Cells were first blocked with TruStain FcX™ PLUS (BioLegend) for 10 minutes then incubated with the following antibodies at 1:200 dilution (BioLegend): PE/Cy7-CD3, PE-CD4, and FITC-CD8. After surface staining, the stained cells were fixed and permeabilized as described in the manufacturer’s instructions (BioLegend) and then stained with APC-IFN-γ or APC-IL-4(1:40, BioLegend). After washing, cell events were acquired using CytoFLEX S (Beckman), and the positive T-cell percentage was analyzed by FlowJo software (FlowJo, LLC).

### Detection of cytokines in the T-cell culture supernatant using ELISA

After the splenic cells had been stimulated with RBD protein for 42 hours, the culture supernatant was harvested for the detection of mouse cytokines *via* ELISA (ELISA MAX™ standard Set Mouse IL-2, ELISA MAX™ standard Set Mouse IL-4, ELISA MAX™ standard Set Mouse IL-10, ELISA MAX™ standard Set Mouse IFN-γ, Biolegend). Due to the limited volume, harvested supernatant from one group was pooled, and three parallel wells were set. The following detection limits were obtained from the manufactures: IL-2 (detection limit 2.0 pg/mL), IL-4 (detection limit 2.0 pg/mL), IL-10 (detection limit 31.3 pg/mL), and IFN-γ (detection limit 15.6 pg/mL). Dilution factors of the samples were determined during a preliminary experiment. The experimental ELISA was carried out according to the manufacturer’s instructions.

### SARS-CoV-2 pseudovirus neutralization

SARS-CoV-2 pseudovirus was prepared as described previously (33). In detail, the genes encoding wild-type or different variants of SARS-CoV-2 with an 18-amino-acid C-terminal truncation were cloned into the pCAGGS plasmid. 293T cells were seeded at a density of 5×10^5^ cells/well in a six-well plate overnight. 293T cells were transfected with these plasmids using Lipo3000 (Thermo Fisher Scientific) and cultured at 37°C with 5% CO_2_. After 24 hours of culturing, the medium was removed, and a volume of VSV△G-EGFP/VSV G (MOI=4) was added to infect the cells for 2 hours, after which the cells were washed with PBS three times. DMEM supplemented with 2% FBS (Gibco) was added. The supernatants containing pseudovirus (VSV△G-EGFP/SARS-CoV-2 S) were collected on the following day and filtered through a 0.45-μm filter. The virus titer was tested in Vero E6 cells.

Gradient diluted BAL samples or serum samples were incubated with an equal volume of 1000 IU (infection unit)/60 μL SARS-CoV-2 pseudovirus at 37°C for 1 hour. The mixture was added to a monolayer of Vero E6 cells in a 96-well plate and incubated for 24 hours. Fluorescence was measured using an Operetta high-content imaging system combined with Harmony imaging and analysis software (PerkinElmer). The inhibition effects of each dilution were evaluated and the half maximal inhibitory concentration was calculated using the Karber method.

### Authentic SARS-CoV-2 neutralization assay

The levels of SARS-CoV-2-specific neutralizing antibodies were determined using PRNT (33) in the BSL-3 facility. Briefly, Vero E6 cells were seeded in 24-well plates at 1.5×10^5^ cells per well, followed by an overnight incubation at 37°C with 5% CO_2_. The serially diluted samples were prepared with DMEM supplemented with 2% FBS and mixed with an equal volume of diluted solution containing 200 TCID_50_/100 μL of wild type SARS-CoV-2 virus. After one hour of incubation at 37°C, the mixtures were transferred to confluent Vero E6 cells. Positive and negative controls were set as cells infected with and without virus. After 1 hour of incubation at 37°C, the antibody–virus mixtures were removed and replaced with 1 mL of DMEM with 2.5% FBS plus 0.9% carboxymethyl cellulose for further incubation at 37°C with 5% CO_2_ for 3 days. After fixation with 4% formaldehyde and crystal violet staining, plaques were counted, and the sample dilution with 50% plaque reduction was calculated as the SARS-CoV-2-specific neutralizing antibody titer.

### hACE2 transgenic mouse vaccination and challenge

Sixteen 4-week-old female hACE2 mice were initially housed and randomly divided into two groups (n=8). Mice in the RBD-mFc group were intranasally immunized with a 10-µg dose of RBD-mFc protein in a 20 μL volume at two-week intervals according to the prime-boost-boost strategy, and PBS vaccination was used as the control. Blood samples were collected 14 days after prime and boost immunization and 12 days after the second booster immunization, and serum was frozen at -80°C. Detection of RBD-specific antibodies was performed to evaluate immunologic effects. On day 12 after the second booster immunization, all mice were transferred to the BSL3 facility. Following anesthesia with 1.25% Avertin (200 mg/kg), mice were challenged intranasally with 1×10^4^ TCID_50_ SARS-CoV-2 in 20 µL DMEM. After challenge, the body weight and survival rate of each group were monitored daily. At 4 days postinfection (dpi), half of the mice in the two groups were euthanized, and the lungs were collected. The lung tissue from each sacrificed mouse was divided into two parts: half of the lung was fixed in 10% buffered formalin and then stored at room temperature and half was weighed, homogenized, frozen and stored at -80°C for future use. The remaining mice were euthanized at 7 dpi.

### Measurement of SARS-CoV-2 genomic RNA in the mouse lung by qRT–PCR

The collected lung samples were weighed and homogenized with 1 mL DMEM in a tissue grinding apparatus and clarified by centrifugation, and the supernatants were aliquoted and stored at -80°C. The viral RNA in mouse lung samples was quantified by one-step real-time quantitative PCR. In detail, 140 µL of lung sample homogenates was subjected to viral RNA extraction using the QIAamp Viral RNA Mini Kit (Qiagen) according to the manufacturer’s instructions. RNA was eluted in 50 µL of elution buffer, and 2 µL of the RNA was used as the template for RT–qPCR to amplify selected genes by using a HiScript II One-step qRT PCR SYBR Green Kit (Vazyme). The following pair of primers targeting the SARS-CoV-2 S gene was used: RBD-qF1: 5’-CAATGGTTTAACAGGCACAGG-3’; RBD-qR1: 5’-CTCAAGTGTCTGTGGATCACG-3’. Amplification was performed as follows: 50°C for 3 min, 95°C for 30 s, and 40 cycles of 95°C for 10 s and 60°C for 30 s in a Step-one real-time PCR system (Bio-Rad). The standard curve was generated using a plasmid encoding the full-length gene of SARS-CoV-2 S protein, and elution buffer was used as the negative control sample. After amplification, the RNA copy number was calculated with the following equation: RNA copies/g tissue = Log10 [(sample cell – negative control well) Ct-converted copies/µL×2 (2 µL of template) ×25 (2 µL of 50 µL total RNA) ×7.14 (140 µL from 1 mL homogenized tissue)/tissue weight (g)].

### Virus detection in lung tissue by plaque assay

For the detection of viral particles in the lungs, the supernatants of lung sample homogenates from the RBD-mFc group and PBS group were diluted at 1:10 and 1:80, respectively. A total of 200 µL diluted supernatant was added to Vero E6 cells in 24-well plates. After 1 hour of incubation at 37°C, the supernatants were removed and replaced with 1 mL of DMEM with 2.5% FBS plus 0.9% carboxymethyl cellulose for further incubation at 37°C with 5% CO_2_ for 3 days. After fixation with 4% formaldehyde and crystal violet staining, plaques were counted. The limit of detection of the plaque assay was approximately 25 pfu/mL.

### Histopathology and immunohistochemistry

The collected lung samples from the two groups (n=4) were fixed in formalin, embedded in paraffin, and cut into 4-μm sections. A portion of the sections were stained with hematoxylin and eosin (H&E), and others were analyzed to detect the SARS-CoV-2 antigen as follows: sections were first blocked with BSA and incubated with a primary antibody (rabbit anti-SARS-CoV-2 NP protein polyclonal antibody at a 1:800 dilution) for 1 hour at 37°C. After washing with PBS, the sections were incubated with Cy3-conjugated goat anti-rabbit IgG followed by incubation with DAPI. The images were collected by a Pannoramic MIDI system (3DHISTECH, Budapest) connected to an FV1200 confocal microscope (Olympus).

### Quantification and statistical analysis

Prism software (version 8, GraphPad) was used for the statistical analysis. For two-group comparisons, a two-tailed unpaired t test was used. For multiple-group comparisons, one-way ANOVA with Tukey’s multiple comparisons test was used. For comparisons among multiple groups with two independent variables, two-way ANOVA followed by Sidak’s multiple comparisons test was used. The log-rank (Mental-Cox) test was used to analyze survival. ns, not significant; *p < 0.05; **p < 0.01; ***p < 0.001; ****p < 0.0001. The mean ± SD was determined for continuous variables as noted. Error bars indicate the standard error.

## Results

### RBD-mFc fusion protein is dimeric, functional and long-lasting in serum

**Figure 1:**
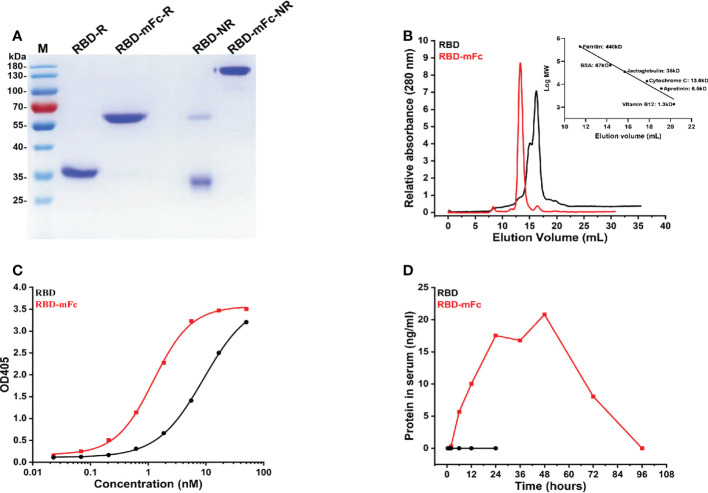
RBD-mFc fusion protein is dimeric, functional and long-lasting in serum. **(A)** Analysis of RBD-mFc and RBD protein (approximately 4 μg each) under reducing and nonreducing conditions through SDS–PAGE (4-12% gradient gel) which indicated that RBD-mFc existed as a disulfide-linked dimer while RBD is a mixture of monomer and dimer. M, marker; R, reduced form; NR, nonreduced form. **(B)** Analysis of RBD-mFc and RBD proteins by size exclusion chromatography on Superdex 200 10/300 (GE). X-axis: elution volume (mL), Y-axis: A280 nm (a.u.) The right inset shows the standard curve. Two peaks were observed in the eluted RBD, while only one peak was separated from the eluted RBD-mFc, indicating that RBD-mFc was uniform. **(C)** Binding of RBD-mFc and RBD to hACE2-Fc, as measured by ELISA. The EC_50_ values of hACE2-Fc binding to coated RBD-mFc and RBD were estimated at 1.2 nM and 9.1 nM, respectively. Data are average values of two replicates. **(D)** Plasma half-life of RBD-mFc and RBD *via* intranasal administration. Purified RBD-mFc and RBD proteins (50 μg) were intranasally inoculated into BALB/c mice (n=3). Serum was collected at the times indicated on the abscissa. The protein concentration in pooled blood circulation were measured by ELISA. Substantial RBD-mFc accumulation and persistence in sera were observed while RBD was barely detectable.

To obtain immunogen for mouse immunization, we generated a recombinant plasmid for the expression of the RBD-mFc fusion protein in mammalian cells by cloning the SARS-CoV-2 RBD in frame with the mouse IgG2a Fc fragment, which is functionally homologous to the human IgG1 Fc fragment (34), according to our previous design (33). The RBD protein alone was also prepared for expression in mammalian cells. The RBD-mFc protein, with a calculated molecular weight of 65 kDa, was a uniform monomer under reducing conditions in SDS–PAGE (**Figure 1A**). Under nonreducing conditions, a dimeric form of RBD-mFc was observed, which was consistent with the results of size exclusion chromatography (SEC) (**Figures 1A, B**). RBD existed mainly as a monomer (~70%) with a minor fraction of dimer (~30%) due to the occasional formation of a disulfide bond between the cysteines at position 538 of the RBD (**Figure 1B**). Functional testing of the recombinant proteins was confirmed by ELISA. The recombinant SARS-CoV-2 receptor protein hACE2-Fc (33) bound to RBD-mFc and RBD coated on plates in a dose-dependent manner, with EC_50_ values of 1.2 nM and 9.1 nM, respectively (**Figure 1C**).

To determine whether recombinant proteins could cross the mucosal barrier and enter the circulation after intranasal inoculation, the purified RBD-mFc and RBD proteins were intranasally administered, and serum was collected at different time points. We observed substantial RBD-mFc accumulation and persistence in the sera for four days, while RBD was barely detectable (**Figure 1D**).

### A strong immune response was elicited by RBD-mFc intranasal immunization

**Figure 2:**
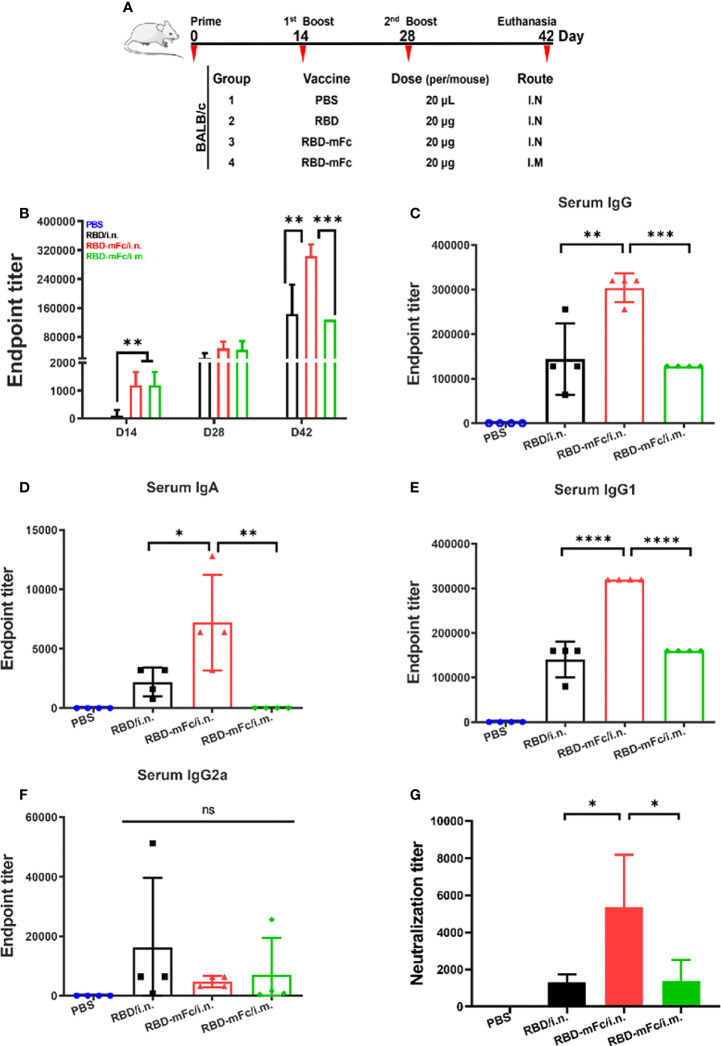
A strong immune response was elicited by RBD-mFc intranasal immunization. **(A)** Schematic of the BALB/c immunization strategy. Four mice from each group were immunized with different vaccines by different routes at the times indicated (days). Serum was collected every two weeks and assessed for specific antibody response to RBD. **(B)** Overall immune response of the four immunized groups. The data show the reciprocal endpoint dilution titers, with each data point representing the mean of four animals. Mice immunized with RBD-mFc protein developed a more rapid and efficient response after prime and boost vaccination compared to RBD. Serum antibody responses were analyzed 14 days after the 2^nd^ boost immunization. RBD-specific IgG **(C)** and IgA **(D)** were assessed by ELISA. Intranasal immunization with RBD-mFc induced a stronger immune effect than immunization in the other two groups. Analysis of IgG subclasses of the RBD-specific antibody response **(E, F)**. **(G)** The neutralizing antibody response to SARS-CoV-2 was determined by PRNT and represented as the reciprocal half-maximal inhibition concentration (IC50). All RBD protein-immunized sera could efficiently inhibit authentic SARS-CoV-2 infection, whereas sera from the PBS control showed no neutralization activity The data in **(B–G)** represent the mean ± SD. Significant differences were determined by one-way ANOVA with Tukey’s multiple comparisons test. ns, not significant; *p < 0.05; **p < 0.01; ***p < 0.001; ****p < 0.0001.

To evaluate the immunogenicity of RBD-mFc *in vivo* (**Figure 2A**), six-week-old BALB/c mice received intranasal immunizations with 20 μg RBD-mFc formulated with alum adjuvant (RBD-mFc/i.n. group). Mice in the RBD/i.n. group were intranasally immunized with an equal protein dose. Meanwhile, mice were intramuscularly vaccinated with the same dose of RBD-mFc (RBD-mFc/i.m. group). A mixture of adjuvant and PBS was designated as the mock treatment. All mice were subjected to another two doses of booster immunizations with an interval of two weeks between each dose. With each round, the antibody response was gradually enhanced in the immunized groups, whereas no specific antibody was detected in the mock group. The magnitude of the IgG response after primary immunization was greater in the two RBD-mFc groups (i.n. and i.m.) than in the RBD group (i.n.), which indicated that the stabilization of the dimeric form of the RBD protein by the Fc fragment has increased potential to elicit a rapid immune response (**Figure 2B**). After the completion of immunization, the mean RBD-specific IgG titer in the RBD-mFc/i.n. group was 3.04×10^5^, which was significantly higher than those in the RBD/i.n. (1.44×10^5^, p=0.0011) and RBD-mFc/i.m. (1.28×10^5^, p=0.0005) groups (**Figure 2C**). Hence, nasal immunization might bring more advantages in the induction of higher total RBD-specific antibody levels (RBD-mFc/i.n. v.s. RBD-mFc/i.m.). The route of administration can substantially modify the quality and magnitude of the immune response (35, 36). Notably, we found only the two i.n. groups exhibited an IgA response (**Figure 2D**). Consistent with the IgG level, RBD-mFc/i.n. elicited a significantly higher IgA titer than RBD/i.n. (p=0.025). The levels of RBD-specific IgG2a and IgG1 subtypes in immune serum, which reflect the Th1- and Th2-type immune response, respectively (37), were also assessed. We found that IgG1 (**Figure 2E**) and IgG2a (**Figure 2F**) subtype antibodies were elicited at appreciable levels among all antigen-immunized mice. Among the three protein-immunized groups, the antibody levels of IgG1 were higher than those of IgG2a because of the use of alum as an adjuvant but also because of the genetic predisposition of BALB/c mice. Previous studies have demonstrated that IgG1 was the major subclass in BALB/c mice (38, 39), which was controlled by Ig allotype-linked genes located on chromosome 12 of different strains of mice (40). The plaque reduction neutralization test (PRNT) was performed to evaluate the neutralizing antibodies (NAbs) in the serum. Immunized mouse serum at 45 days post-primary vaccination strongly inhibited authentic SARS-CoV-2 infection. The titer of 50% plaque reduction in the RBD-mFc/i.n. group (4425) was considerably higher than those in the other groups (1267 in RBD/i.n. and 1071 in RBD-mFc/i.m. p=0.0416, p=0.0222, respectively) (**Figure 2G**). These results demonstrated that intranasal immunization of the bivalent RBD (RBD-mFc/i.n.) vaccine elicited an enhanced response compared to monomer (RBD/i.n.) and intramuscular immunization (RBD-mFc/i.m.), which indicates the benefits of using Fc as a carrier for intranasal vaccination.

**Figure 3:**
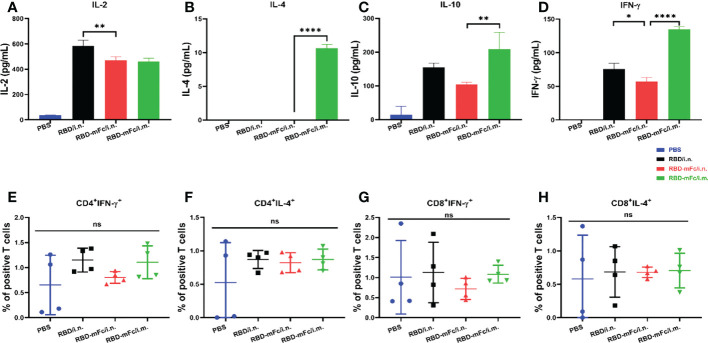
Cellular response induced by RBD-mFc intranasal immunization. Vaccinated BALB/c mice were euthanized at 2 weeks post-2nd boost immunization. The splenocytes harvested from immunized mice (n=4) were stimulated with RBD protein for 42 hours. Cultured supernatants were harvested and pooled. Cytokine production was measured using an ELISA kit. RBD-immunized mice generated a certain amount of IL-2 **(A)**, IL-10 **(C)** and IFN-γ **(D)**. IL-4 **(B)** was detected only in RBD-mFc intramuscularly immunized mice. Data represent the mean of three parallel wells in one experiment. Splenocytes were incubated with SARS-CoV-2 RBD protein. The responding CD4+ **(E, F)** and CD8+ T **(G, H)** cells were identified by intracellular staining for effector cytokines. The gating strategies are shown in **Supplementary Figure S1**. Data are represented as the mean ± SD. Significant differences were determined by one-way ANOVA with Tukey’s multiple comparisons test. ns, not significant; *p < 0.05; **p < 0.01; ****p < 0.0001.

The T-cell response has been shown to be important for viral clearance during SARS-CoV-2 infection and may be decisive for potential cross-reactive protection against variants *in vivo* (41, 42). To confirm whether intranasal administration of RBD-mFc could effectively elicit a cellular response, two methods were applied to detect the SARS-CoV-2 response. Intracellular cytokine staining (ICS) assays were used to assess RBD-specific CD4^+^ and CD8^+^ T cells. Cytokine ELISA analysis was utilized to determine the degree of the RBD-specific T-cell response. Briefly, two weeks after the second boost immunization, mice were euthanized, and spleens were harvested to obtain splenocytes. Flow cytometry analysis (**Figure S1**) of splenic cells stimulated with RBD protein indicated that the levels of both IFN-γ and IL-4 produced by T cells among all immunized groups, including the PBS group, were not significantly different (**Figures 3E–H**, **S2**). After *in vitro* antigen stimulation, IL-2, IL-10 and IFN-γ levels were increased and were higher in the total splenic T-cell culture supernatant from three protein-immunized group mice than in the PBS group (**Figures 3A, C, D**), while very low levels of IL-4 were detected only in RBD-mFc/i.m. (**Figure 3B**). Among the two distinct vaccination routes, it seemed that T cells obtained from the intramuscular immunized group generated more IL-4 and IFN-γ than those obtained from the intranasal group, which indicated that intramuscular vaccination induced stronger cellular immunity in the spleen than the intranasal route. Therefore, an effective mucosal immune adjuvant for intranasal vaccination should be applied in the future to enhance the cellular immune response (43).

### Broader neutralizing antibodies against SARS-CoV-2 variants were induced by RBD-mFc intranasal immunization

The rapid emergence of SARS-CoV-2 VOCs has seriously challenged approved immune interventions. The VOCs that have been designated to date are Alpha (B.1.1.7), from the United Kingdom (44); Beta (B.1.351), from South Africa (45); Gamma (P.1), from Brazil (46); Delta (B.1.167.2), from India (47) and Omicron (B.1.1.529), from South Africa (48). Among these VOCs, Omicron exhibits significant evasion of the protection elicited by currently approved vaccines and has gradually become dominant (49–51).

**Figure 4:**
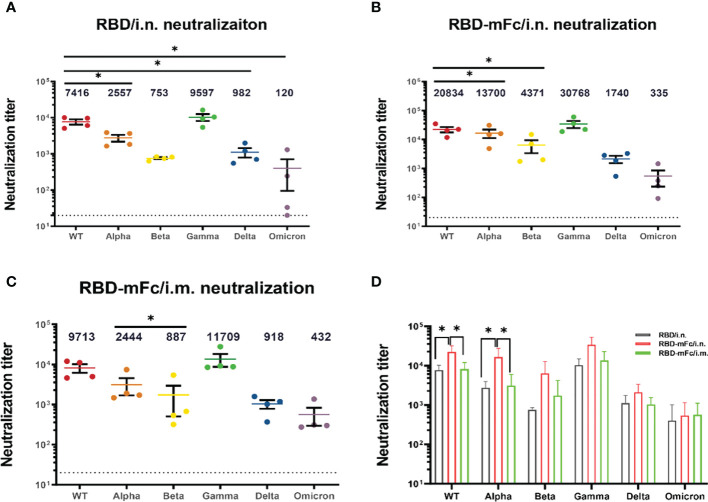
Broader neutralizing antibodies against SARS-CoV-2 variants were induced by RBD-mFc intranasal immunization. The cross-reactive neutralization of immune sera against wild-type and variants of SARS-CoV-2 was analyzed by pseudovirus neutralization. **(A–C)** Neutralization titers for wild-type and VOC pseudoviruses generated by immune sera from the **(A)** RBD/i.n., **(B)** RBD-mFc/i.n. and **(C)** RBD-mFc/i.m. groups 14 days after the 2^nd^ boost immunization. The majority of immune sera showed broad-spectrum neutralization capacity. All groups n=4. The dotted lines represent the limit of detection (1:20 dilution). Geometric mean titers calculated by GraphPad Prism are shown above each column. **(D)** Comparison of neutralization titers against individual viruses between immune sera from the RBD/i.n., RBD-mFc/i.n. and RBD-mFc/i.m. groups. Data are presented as the mean ± SD; nonparametric ANOVA with Dunn’s multiple comparison test was used to test for significant differences in **(A)**. Significant differences in **(B–D)** were determined by one-way ANOVA with Tukey’s multiple comparisons test. *p < 0.05.

We applied a pseudovirus neutralization assay to confirm the cross-neutralizing effectiveness of immune sera from RBD/i.n., RBD-mFc/i.n. and RBD-mFc/i.m. against wild-type SARS-CoV-2 and the alpha, beta, gamma, delta and omicron variants. All immunized mouse sera showed slightly enhanced neutralizing ability against the gamma variant and reduced activity against the remaining four variants compared with that against the wild-type viral strain (**Figures 4A–C**). The neutralization titers of RBD-mFc/i.n. group against the WT and alpha variants were significantly higher than those of the other two groups (**Figure 4D**). Although the neutralization titer among all groups was dramatically lower for the Omicron variant than for the wild-type virus, immune sera from RBD-mFc-immunized mice maintained neutralizing activity (IC_50 =_ 335) against the Omicron variant. In the RBD/i.n. group, three of the four mouse sera neutralized the omicron variant with low ability. For wild-type SARS-CoV-2 and the Alpha variant, intranasal immunization with RBD-mFc induced higher neutralization antibody levels. However, Fc-fusion proteins induced stronger humoral immunity against VOC than RBD protein regardless of vaccine route. In general, immune sera from RBD-mFc-immunized mice (i.n.) have higher neutralizing titers than RBD-immunized mice. Moreover, intranasal immunization with RBD-mFc induced a stronger humoral immune response than intramuscular immunization. These results indicate the advantages of fusion with the Fc fragment and benefits from intranasal immunization. Taken together, these results indicate that intranasal immunization with RBD-mFc could elicit broadly neutralizing antibodies against different VOCs.

### Robust mucosal immunity in the respiratory system was induced by RBD-mFc *via* intranasal immunization

**Figure 5:**
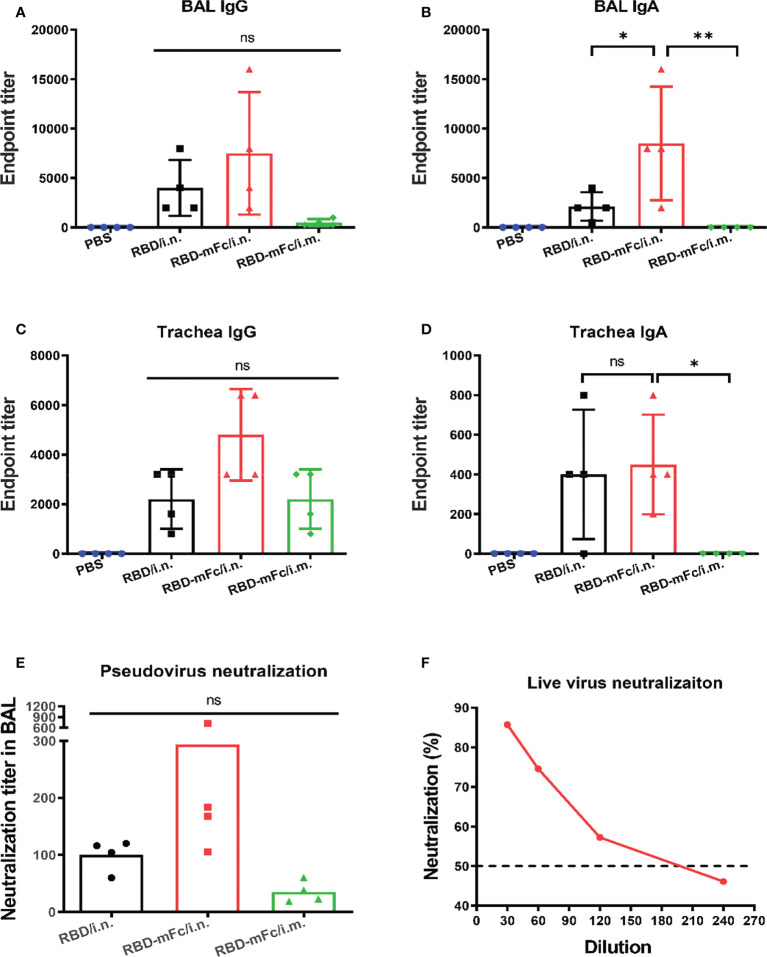
Robust mucosal immunity in the respiratory system was induced by RBD-mFc *via* intranasal immunization. The fully immunized mice were euthanized 14 days after the 2^nd^ boost immunization, and samples from the respiratory tract, including bronchoalveolar lavage fluid (BAL) and trachea wash, were collected. **(A–D)** The antibody response in these samples were determined by evaluating RBD-specific IgG and IgA. In contrast to intramuscular vaccination, intranasal immunization could additionally induce mucosal immunity. **(E)** The neutralization titer in BAL fluid were determined by a SARS-CoV-2 pseudovirus neutralization assay. **(F)** Neutralization of authentic SARS-CoV-2 virus by pooled BAL from RBD-mFc/i.n. group. The data in **(A–D)** represent the mean ± SD. Significant differences were determined by one-way ANOVA with Tukey’s multiple comparisons test. ns, not significant; *p < 0.05; **p < 0.01.

Secreted IgA at the mucosal site is an important partner of the immune response against respiratory pathogens, especially SARS-CoV-2 (52). It is necessary to evaluate the capacity of RBD-mFc to induce mucosal immunity. The fully immunized mice were euthanized at Day 42, and samples from the respiratory tract, including bronchoalveolar lavage fluid (BAL), nasal wash and tracheal wash, were collected and analyzed to measure the local immune response (**Figure 5**). In all antigen-immunized groups, obvious levels of IgG were detected in all tested samples (**Figures 5A, C**; **S3A**). The IgG responses in RBD-mFc/i.n. were slightly higher those of the other two groups, although there was no statistically significant difference. Notably, only intranasal immunization of RBD protein (RBD-mFc/i.n. and RBD/i.n. groups) could induce detectable IgA in BAL and trachea, while no IgA was induced in the intramuscular immunization group (RBD-mFc/i.m.) in all test samples **(** **Figures 5B, D**). We also observed that one mouse each in the RBD/i.n. and RBD-mFc/i.n. groups had IgA in the nose, possibly due to individual differences (**Figure S3B**). RBD-mFc significantly improved the production of IgA in lung by 3-fold compared with RBD (6727 vs. 1682).

Simultaneously, we sought to determine the level of NAbs in the lung. Pseudovirus-based inhibition assays showed that pooled BAL in the RBD-mFc/i.n. group neutralized SARS-CoV-2 pseudovirus with a higher neutralization titer (IC_50 =_ 294) than RBD-mFc/i.m. (IC_50 =_ 35). In contrast, the titer of BAL in the RBD/i.n. group showed a modest value of 100 (**Figure 5E**). To further validate the neutralization capacity of BAL from RBD-mFc/i.n. group against authentic virus, we performed a PRNT with authentic SARS-CoV-2. The PRNT_50_ titer was 173 (**Figure 5F**), which was consistent with the results of the pseudovirus assays (**Figure 5E**). In general, mice in the RBD-mFc/i.n. group developed higher antibody titers in the respiratory tract than mice in the other two groups.

### A low dose of RBD-mFc could induce a lasting and strong immune response

**Figure 6:**
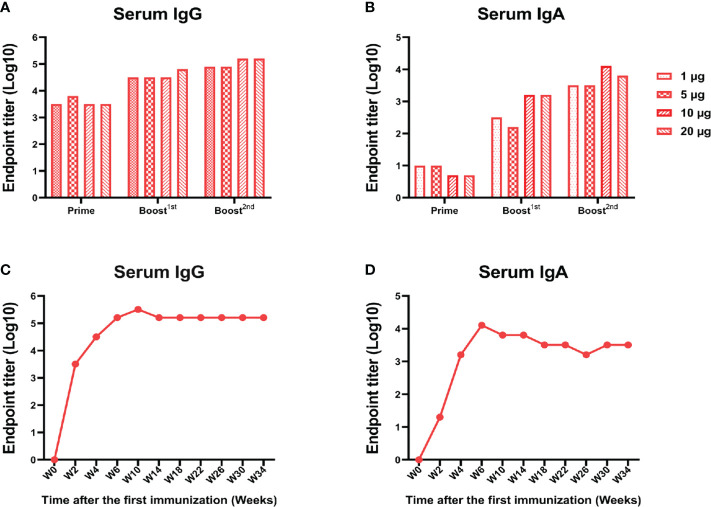
A low dose of RBD-mFc induced a lasting and strong immune response.BALB/c mice (n=4) were intranasally immunized with different doses of RBD-mFc. Serum was collected and pooled every two weeks after the prime immunization. RBD-specific IgG **(A)** and IgA **(B)** were assessed by ELISA. Increasing antibody responses were induced by increasing the immune cycle in a dose-dependent manner. To test for antibody persistence after immunization, a group of 10 μg immunized mice was chosen to assess RBD-specific IgG **(C)** and IgA **(D)** duration. Antibodies in serum underwent dynamic change and plateaued until the end of detection.

In view of the above finding that RBD-mFc intranasal immunization could induce a potent humoral response, we further explored whether this response was dose dependent. BALB/c mice were immunized with RBD-mFc at 1, 5, 10, or 20 μg. At 14 days after primary immunization, 100% seroconversion was detected in all groups, and we also observed a robust anti-RBD IgG response to boost immunization (**Figure 6A**). At the completion of the immunization schedule, the RBD-specific IgG titer in sera reached 10^5^ in both the 10 and 20 μg dose groups, which was higher than the titers in the 1 and 5 μg dose groups (**Figure 6A**). The IgA response was also obvious. Notably, the 10 μg dose induced a higher IgA titer than the other doses (**Figure 6B**). The duration of protective immunity elicited by vaccination is also crucial for evaluation of a vaccine. We found that the RBD-specific IgG and IgA titers in mouse sera after completion of the 10 μg dose vaccination could be maintained for more than 210 days (**Figures 6C, D**). These results suggested that RBD-mFc could induce long-term humoral and mucosal responses *via* intranasal immunization.

### RBD-mFc protected mice from lethal challenge with SARS-CoV-2

**Figure 7:**
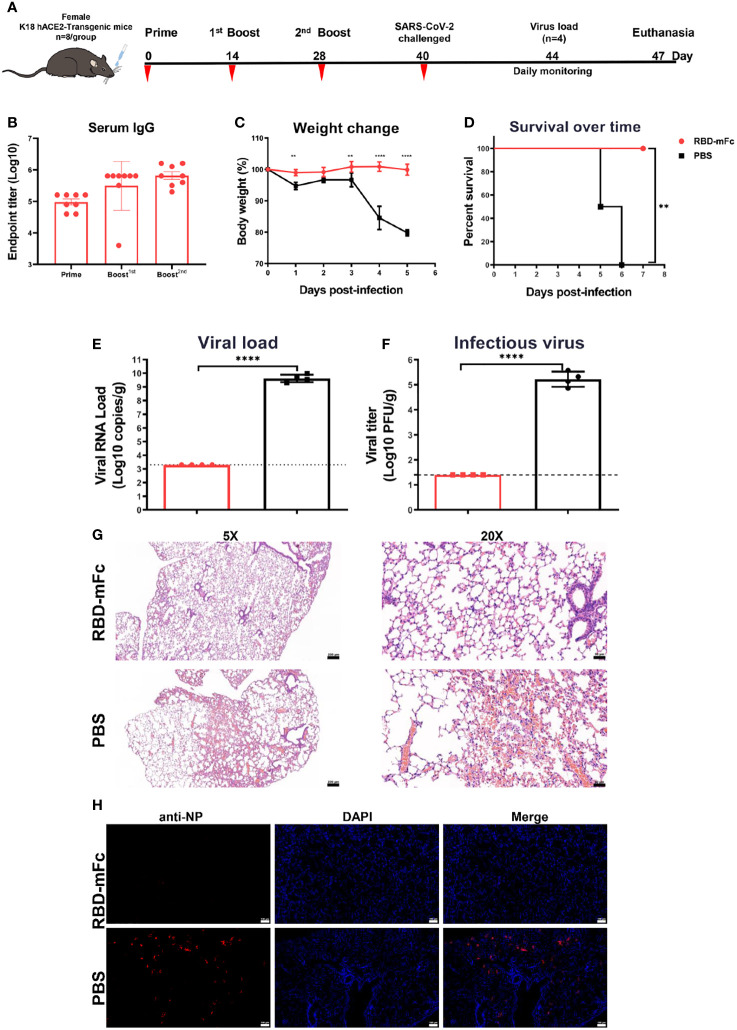
RBD-mFc protects mice from lethal challenge with SARS-CoV-2. **(A)** Immunization and challenge schedules for hACE2 transgenic mice. Female hACE2 transgenic mice (n=8) were intranasally immunized with 10 μg RBD-mFc protein on days 0, 14 and 28. Equal volumes of adjuvant mixed with PBS were used as controls. Blood samples were collected at the times indicated. All vaccinated mice were challenged with 20 μL of 1×10^4^ TCID_50_/mouse wild-type SARS-CoV-2. Half of the mice were sacrificed at day 4 postinfection to assess the viral load; the remaining mice were monitored until 7 days postinfection. **(B)** Overall RBD-specific antibody response after the prime immunization. A potent antibody response was successfully induced in all mice. **(C)** Body weight change of mice for 5 days. **(D)** Survival curve. Mice in the PBS group experienced a shaped weight change at 4 dpi and gradually started to die. In the RBD-mFc group, the mice were well maintained. SARS-CoV-2 RNA copies detected by RT‒qPCR **(E)** and titers of infectious viral particles assessed by plaque assay **(F)** at 4 dpi in homogenized lung. A large amount of virus was detected in the control group, while all detected samples from the RBD-mFc group were under the detection limit. HE staining **(G)** and IFA against N protein **(H)** were evaluated in lungs at 4 dpi. No observed in the RBD-mFc group compared with the PBS group. The scale bar in **(G, H)** represents 100 μm. Data are represented as the mean ± SD. Significant differences in **(C)** were determined by two-way ANOVA with Sidak’s multiple comparisons test. Data in **(D)** were analyzed by the log-rank (Mantel‒Cox) test. Significant differences in **(E, F)** were determined by a two-tailed unpaired t test. **p < 0.01; ****p < 0.0001.

K18-hACE2 mice are a lethal murine model for SARS-CoV-2 with infectious features in the lung that are similar to those of severe human COVID-19 (53, 54).To further evaluate the protective efficacy against SARS-CoV-2 *in vivo*, we routinely intranasally immunized transgenic hACE2 mice three times with 10 μg RBD-mFc protein formulated with alum adjuvant (n=8) and PBS as a control (n=8) (**Figure 7A**). In the RBD-mFc group, RBD-specific IgG antibody titers increased over time following the initial primary immunization, with substantial boosting observed after the two subsequent immunizations. The average titers were 4-fold higher than those in the normal BALB/c mice, with levels of approximately 6×10^5^ (**Figure 7B**). The immunized mice were challenged by intranasal inoculation with 1×10^4^ TCID_50_ of wild-type SARS-CoV-2 on Day 40 post primary immunization. Half of the mice were monitored daily for body weight change and morbidity for 7 continuous days; the remaining half were sacrificed at day 4 postinfection, and their lungs were collected for analysis.

Following the challenge, all RBD-mFc-immunized mice survived without significant body weight loss (**Figures 7C, D**). In contrast, the mice in the PBS group exhibited sharp body weight loss, which was pronounced at 4 dpi, and began to die on day 5 with increasing mortality up to 100% at 6 dpi (**Figures 7C, D**). The viral load in the lung tissues was measured by both qRT–PCR and plaque assays. Very high levels of SARS-CoV-2 RNA copies (1.9×10^8^ copies/mL) and infectious virus titers (10^5^ PFU/mL/g) were detected in the PBS group, whereas samples in the RBD-mFc group tested negative, with both viral RNA and infectious virus titers below the limits of detection (all p<0.0001, **Figures 7E, F**). Histopathological analysis of the lungs of mice in the PBS group (**Figure 7G**) revealed marked pulmonary pathology associated with SARS-CoV-2 infection, characterized by infiltration of inflammatory cells into the interstitium and alveoli, thickened alveolar walls and congested large vasculature. In contrast, vaccination eliminated such disease manifestation, with all the lungs in the RBD-mFc group showing no essential lesions. Immunofluorescence assays were performed to evaluate the presence of SARS-CoV-2 viral antigens in infected lungs. Strongly positive SARS-CoV-2 cells were densely distributed in control group lungs, while no viral antigen staining was observed in vaccinated animals (**Figure 7H**). Overall, intranasal immunization with RBD-mFc was able to provide protection with complete clearance of the virus in the lungs.

## Discussion

The upper respiratory tract is the primary site of SARS-CoV-2 infection (55). The virus initially infects epidermal cells in the nasopharynx and subsequently spreads to other ACE2-expressing epidermal cells in the lung. However, the majority of approved vaccines against SARS-CoV-2 are delivered by intramuscular injection, which elicits a systemic IgG response but does not effectively evoke mucosal immunity (such as IgA), especially in the respiratory tract. These limitations might reduce the protective efficacy of these vaccines. In fact, IgAs are produced earlier than IgGs and dominate neutralizing antibodies in the protective response of COVID-19 patients during the early stage of infection (52). Therefore, an intranasally administered mucosal vaccine that can induce a high-level response of IgAs in the respiratory tract is highly desired (37, 56). The intranasal administration of the vaccine mimics natural respiratory virus infection and rapidly induces IgA production in the nasal cavity, which is actively transported across the epithelium at nasal passages and released in respiratory fluids in the lumen to obstruct viral entry. In addition, IgA, as a dimer joined by a J chain, was shown to be more stable and might provide enhanced neutralization and cross-reactivity compared with IgG (57). The induction of secretory IgA has been shown to be associated with resistance to various pathogen infections, mainly through inhibition of viral entry into epithelial cells, mediation of pathogen excretion and prevention of viral particle assembly (58, 59). Several mucosal vaccines have shown robust systemic and mucosal immunity (60, 61). RBD-Fc, as an ideal immunogen, has already been evaluated *in vivo* in other studies, which showed that intramuscular vaccination with RBD-Fc could induce potent humoral immunity (62, 63). In our study, we further evaluated the potential of the RBD-Fc fusion protein as a candidate subunit vaccine delivered intranasally. It is clear that RBD-mFc/i.n. evokes not only a higher humoral response than RBD/i.n. and RBD-mFc/i.m. but also robust mucosal immunity, with a high level of secretory IgA distributed in the upper respiratory tract, which indicates the potential of intranasally administered RBD-mFc as a mucosal vaccine to provide better protective efficacy. We also noted a less pronounced difference between RBD-mFc/i.m. and RBD-mFc/i.n. for IgG than for IgA in the mucosal site, presumably because serum IgG could be transported from the circulation to the mucosal surface of the respiratory tract, whereas IgA is strictly associated with mucosal immunity generation (61).

Cell-mediated immunity to SARS-CoV-2 also plays an important role in viral clearance and protection against evolving variants. In our study, the amounts of IL-2, IL-10 and IFN-γ in the three protein-vaccinated mouse groups were increased compared to those in the PBS group. Notably, intramuscular immunization with RBD-mFc induced relatively higher levels of IL-4 than immunized with RBD/i.n. and RBD-mFc/i.m., suggesting that mucosal immunization with alum adjuvant was not preferential for the induction of a strong cellular immune response. We also found that in line with other studies (64), when compared to other cytokines, the level of IL-4 was low. For protein vaccines, the induction of cellular immunity typically depends on the adjuvant, which could be optimized (65). We also noted that IFN-γ levels in the three protein-immunized groups increased after RBD stimulation compared to PBS in the ELISA, while no significant difference was observed in the intracellular cytokine assay. This finding may in part be explained by the low sensitivity of flow cytometry compared to ELISpot (66, 67). Previous studies have shown that intranasal immunization induces weaker splenic T-cell responses than those induced following intramuscular immunization (60, 68). In our study, the frequency of RBD-specific positive T-cells was too low to allow us to unambiguously judge the cellular response, while the detection of cytokines in the cultured splenocyte supernatant indicated that intramuscular immunization indeed elicited stronger cellular immunity than intranasal delivery. However, due to the relatively small size of animals in the experiment, we cannot draw definite conclusions. The immune response in the lung and BAL may be more informative fro assessing the cellular immunity of mucosal vaccines. Aluminum salt, the most commonly used adjuvant, mainly induces humoral responses and Th2-biased cell responses in mice (69). However, in a study of vaccines against several viruses, Th2-directed cellular immunity resulted in the induction of antibody-dependent enhancement (ADE). There have been no reports of ADE in preclinical or clinical research on Th2-biased SARS-CoV-2 vaccines. In our study, aluminum induced not only IgG1 (Th2) but also IgG2a (Th1) responses. To achieve Th-balanced cellular immunity and enhance mucosal immunity, the optimization of immunoadjuvants should be explored in our next step.

A number of studies have focused on the design of RBD dimers as immunogens *via* intramuscular vaccination (62, 70, 71). Here, we used Fc to achieve dimerization of the SARS-CoV-2 S protein RBD. Fc has been widely used in the construction of many viral immunogens as candidate vaccines, including human immunodeficiency virus-1 (HIV-1) (72), respiratory syncytial virus (RSV) (73), herpes simplex virus-1 (HSV-1) (32), Ebola virus (EBOV) (74) and influenza virus (37). The Fc fragment could stabilize the fusion protein, extend its serum half-life, and serve as an immunopotentiator to enhance immunogenicity, especially for mucosal immunity. We presume that RBD-mFc crossed the respiratory barrier to the bloodstream *via* the interaction of the Fc domain with its neonatal Fc receptor (FcRn), thus also extending the serum half-life of the protein (75). With the aid of FcRn-mediated transcytosis, Fc fusion protein administered intranasally could be rapidly delivered to lymphoid organs *via* the circulatory system that activated humoral immunity with induction of mucosal immune response (72). Collectively, these data demonstrate the promising potential of RBD-mFc as a vaccine candidate. The mechanism of mucosal immunity induced by Fc-fusion protein needs additional research in the future.

Homologous and heterologous booster vaccination is currently ongoing to overcome the reduced vaccine efficacy against SARS-CoV-2 VOCs, while next-generation vaccines are also under development. To date, extraordinary efforts to develop effective vaccines against the SARS-CoV-2 pandemic are mainly based on Omicron and its sublineage variants. According to our studies, the intranasal vaccine RBD-Fc based on Omicron variants, with better safety and user friendliness, could be a good candidate for providing greater protection against the current epidemic and future SARS-CoV-2 variants.

## Data availability statement

The original contributions presented in the study are included in the article/**Supplementary Material**. Further inquiries can be directed to the corresponding author.

## Ethics statement

The animal study was reviewed and approved by the Institutional Review Board of the Wuhan Institute of Virology, Chinese Academy of Science.

## Author contributions

RG conceived and coordinated the study. LC, HZ, ML, BW and ZZ performed the experiments and data analysis. LC and RG wrote the initial draft. RG provided the final approval of the manuscript. All authors contributed to the article and approved the submitted version.

## Funding

This work was jointly funded by the CAS-VPST Silk Road Science Fund 2021 (GJHZ2021134), the SARS-CoV-2 emergency project of the Wuhan Science and Technology Bureau (2020020101010001), the Natural Science Foundation of Hubei Province of China (2019CFA076), and the National Natural Science Foundation of China (32170949).

## Acknowledgments

We thank the staff from the Institutional Center for Shared Technologies and Facilities of Wuhan Institute of Virology. We are grateful to the BSL-3 laboratory of Wuhan Institute of Virology for technical support and their help during the work. We thank the National Virus Resource Center for resource support.

## Conflict of interest

The authors declare that the research was conducted in the absence of any commercial or financial relationships that could be construed as a potential conflict of interest.

## Publisher’s note

All claims expressed in this article are solely those of the authors and do not necessarily represent those of their affiliated organizations, or those of the publisher, the editors and the reviewers. Any product that may be evaluated in this article, or claim that may be made by its manufacturer, is not guaranteed or endorsed by the publisher.
