# Supplementary material for: An intranasal vaccine targeting the receptor binding domain of SARS-CoV-2 elicits a protective immune response
Source: Front Immunol. 2022 Nov 16;13:1005321. doi: 10.3389/fimmu.2022.1005321 (PMC9708728; doi:10.3389/fimmu.2022.1005321)
Supplement: Supplementary file 1 [file DataSheet_1.docx]

**
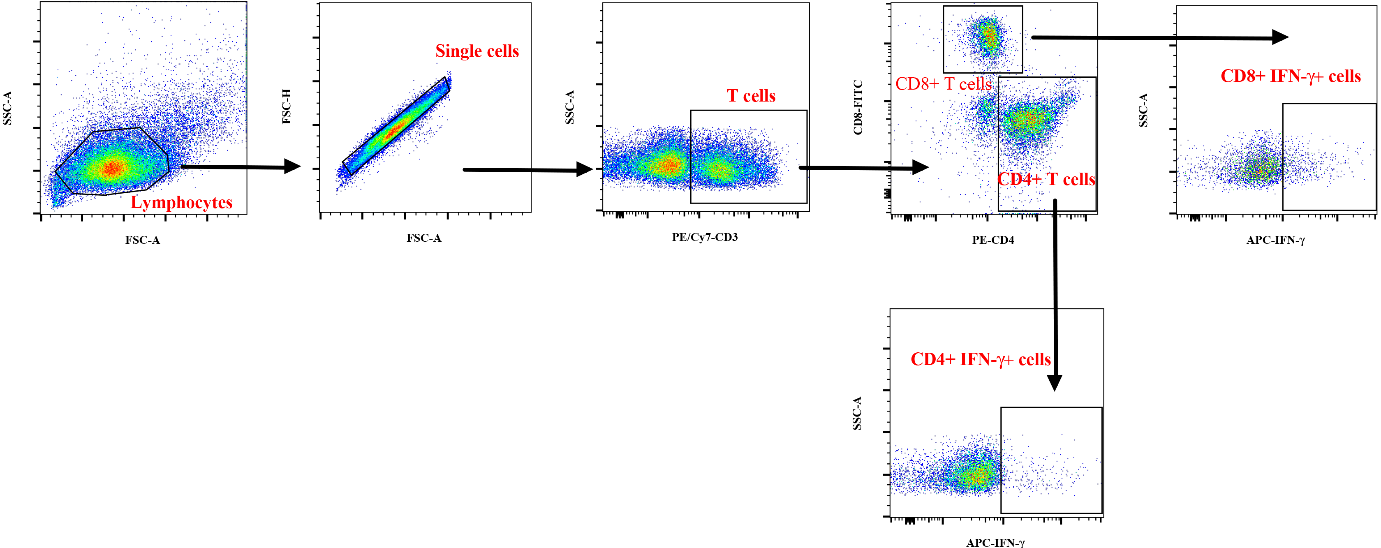
**

**Figure S1. Gating strategy of flow cytometric analysis.**

Mononuclear cells were gated out of total events followed by subsequent singlet gating. CD3^+^ T cells were then divided into CD4^+^ or CD8^+^ T cells. The next gating strategy was to analyze the percentage of IFN-γ^+^ in different T-cell subsets. The analysis of IL-4^+^ T cell subsets was performed using the same scheme.


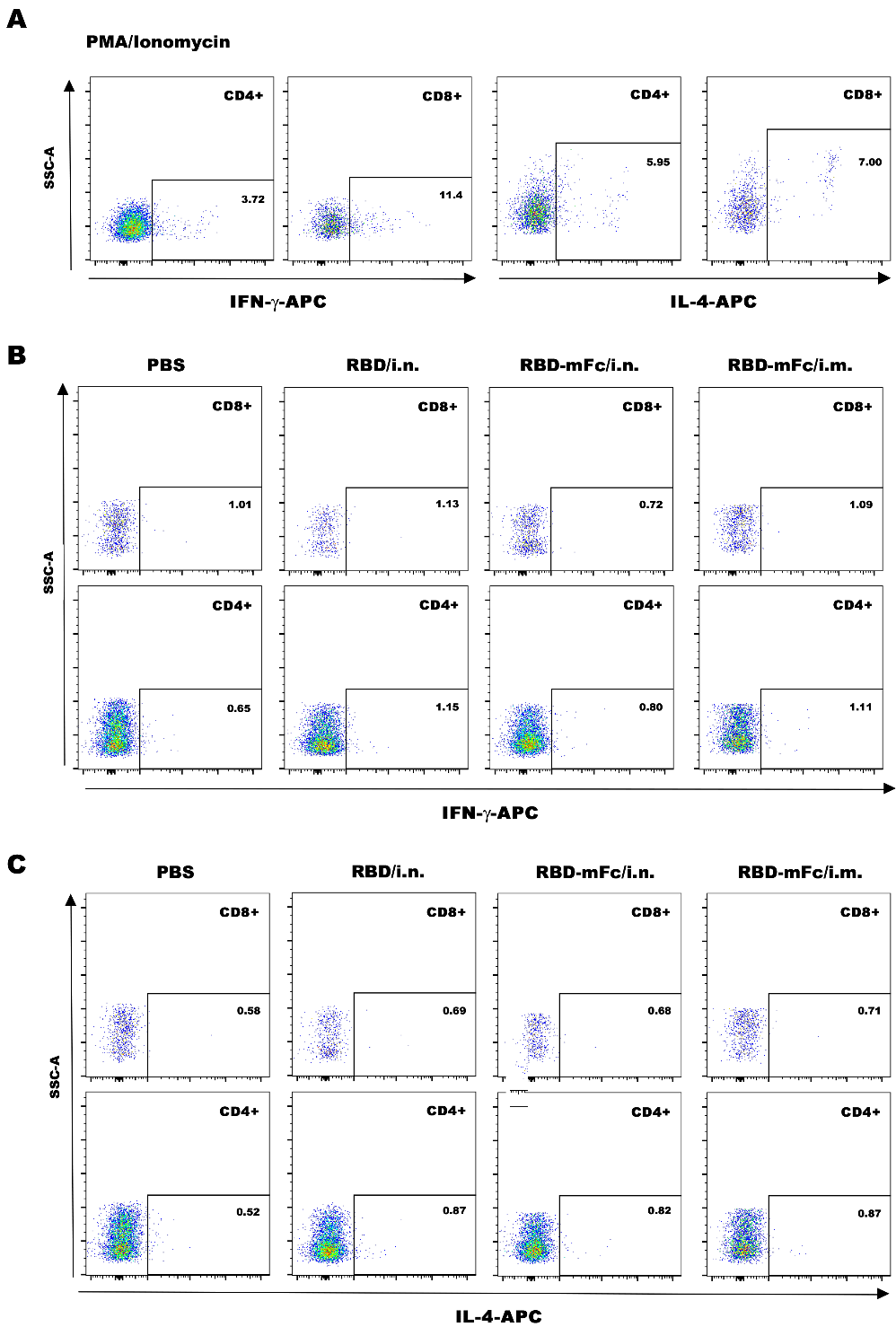


**Figure S2. Flow cytometric dot plots of IFN-γ^+^ and IL-4^+^ T cells from spleens at two weeks post immunization.**

BALB/c mice were euthanized at 2 weeks post-2^nd^ boost immunization. Splenocytes were incubated with SARS-CoV-2 RBD protein, and PMA/ionomycin was used as a positive control. (**A**) The frequency of positive T cells after stimulation with PMA/ionomycin. The frequency of IFN-γ^+^ (**B**) and IL-4^+^ (**C**) positive T cells after RBD protein stimulation.

Data represented in (**B** and **C**) represent the mean. Data are representative of one independent experiment. n=4 mice per group.


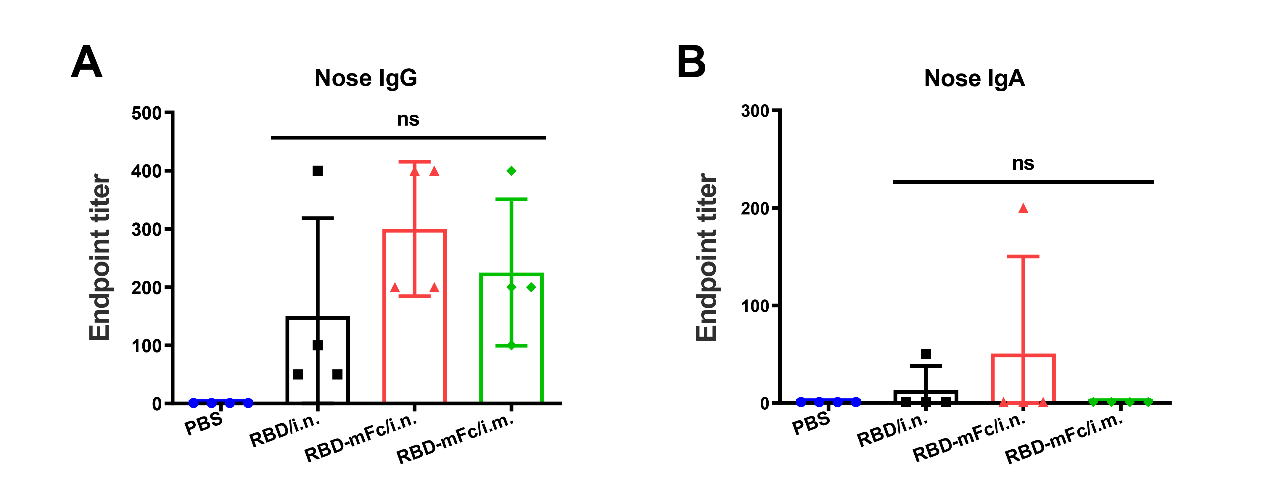


**Figure S3. Immune response in nose induced by RBD-mFc intranasal immunization.**

The fully immunized mice were euthanized at Day 42, and nasal wash samples were collected. The antibody response in these samples was determined by evaluating RBD-specific IgG and IgA. Data are represented as the mean ± SD. Significant differences were determined by one-way ANOVA with Tukey’s multiple comparisons test. ns, not significant.
